# Magnetic Resonance Imaging Evaluation of Pregnancy and Aging on External Anal Sphincter Volume in Squirrel Monkeys

**DOI:** 10.1007/s00192-025-06189-9

**Published:** 2025-06-30

**Authors:** Christopher P. Chung, Wilma I. Larsen, Thomas J. Kuehl

**Affiliations:** 1 Division of Gynecologic Oncology, City of Hope Medical Center, Duarte, CA USA; 2 The Joint Commission, Chicago, IL USA; 3 Artemis Biotechnologies LLC, Austin, TX USA

**Keywords:** External anal sphincter, Magnetic resonance imaging, Squirrel monkey

## Abstract

**Introduction and Hypothesis:**

Both humans and squirrel monkeys are susceptible to pelvic floor injury and prolapse from pregnancy, delivery, and aging. The mechanisms for external anal sphincter (EAS) injury in squirrel monkeys have not been evaluated in detail. This study evaluates a method for measuring EAS volume using magnetic resonance imaging (MRI) in squirrel monkeys and demonstrates the feasibility of serial measurements.

**Methods:**

Using a previously described procedure, MRI was performed on 10 squirrel monkeys prior to euthanasia. After euthanasia, tissue blocks of EAS were cryo-sectioned and stained with succinic dehydrogenase to identify EAS striated muscle fibers. EAS volumes from both MRI and histological measurements were calculated using image analysis software. MRI measurements were obtained by two independent investigators. A cohort of four monkeys, each having five pregnancies and 11 MRI studies over a 5-year span, was used to evaluate EAS volume changes serially within females.

**Results:**

Volumes measured by MRI for each squirrel monkey were similar for the two researchers (Cronbach alpha of 0.97 with 95% lower confidence limit of 0.92), and they were statistically consistent with the volumes obtained from analysis of histology (linear regression with R-squared of 0.97 and *p* < 0.0001). This validated technique was used to measure EAS volumes in four breeding females and demonstrated sufficient power to detect a decrease (*p* < 0.00001) from 32.1 ± 3.2 mm^3^ (mean ± SE) prior to the first pregnancy to 10.7 ± 1.5 mm^3^ after five pregnancies in 5 years.

**Conclusion:**

Magnetic resonance imaging is a valid technique for measuring EAS volume changes in squirrel monkeys with sufficient sensitivity to detect EAS volume changes such that effects of aging and serial pregnancies can be evaluated.

## Introduction

The female squirrel monkey is relatively unique among nonhuman animal species in that it spontaneously develops pelvic organ prolapse (POP) in response to parturition and aging that resembles that of women in terms of degree and frequency [1, 2]. Previously, we described key pelvic floor muscles and their innervation along with connective tissue elements of the vaginal wall using imaging and histology of fixed tissues [4–6]. We also described the impact of nerve injury, parturition, and aging on pelvic floor muscles and connective tissue [8–10] and performed a prospective trial of the effects of cesarean section using this animal as a model species [12]. Some of this work described innervation of the external anal sphincter (EAS) using nerve-tracing methods [5]. In preliminary studies, we also reported on EAS muscle fiber types and potential for variation in relation to POP [13]. However, these histological methods do not allow serial measurements within animal subjects. We are interested in EAS injury in this species, because of a well-established association of EAS injury with pregnancy, delivery, and aging in humans [14]. As there is the potential for planned cesarean section to reduce pelvic floor injury, a validated method could extend prospective randomized trials to include EAS volume assessments.

Although EAS injury in squirrel monkeys has not been evaluated, magnetic resonance imaging (MRI) has been used to measure pelvic floor muscle volumes in female squirrel monkeys to describe changes related to pregnancy, parturition, and aging [8, 9, 13]. A histological method for the measurement of EAS muscle mass has been described in a rat model of childbirth injury [15]. Denervation and re-innervation of the EAS after childbirth can lead to alteration in muscle function that may contribute to incontinence [16]. Muscle-volume alteration is one indicator of such injury.

Harvesting the actual muscle in question from a euthanized animal for histological analysis allows precise measurements of muscle characteristics; however, such methods do not allow for serial tracking of changes in muscle over time. MRI is preferable for this reason, but researchers and physicians need to have confidence in its reliability. Therefore, this study had two objectives: to validate MRI as a method for measuring EAS volume in squirrel monkeys and to test the hypothesis that serial measures in small sample sizes are sufficient to detect altered EAS volumes.

## Materials and Methods

### Study Population

Squirrel monkeys were obtained from the National Squirrel Monkey Breeding and Research Center and housed at our Animal Facility. Our Institutional Animal Care and Use Committee approved the use of the animals in these studies. Two groups of animals were used. One group was a cohort of ten females with variable ages and obstetric histories for validation of techniques. A second group of four females was followed with serial MRI sessions for 5 years in relation to pregnancy and parturition. Members of this latter cohort have been previously studied using serial MRI to observe changes in the pelvic floor muscles in relation to pregnancy, delivery, and aging [10]. However, the series evaluated in the current study represented more pregnancies and was focused on the measurement of EAS volume.

Squirrel monkeys are seasonal breeders that do not menstruate. Females reach puberty between 2.5 and 3.5 years and full adult size by 5 years of age. The peak of their reproductive season is between December and April in the northern latitudes. The cohort of breeding females observed in this study was between 4 and 5 years of age at the time of conception of their first pregnancy. Images were made before or early in the pregnancy, which was diagnosed and monitored by ultrasound, as were all subsequent pregnancies using methods previously described [12].

### Magnetic Resonance Imaging

Animals assigned to this project underwent MRI according to the protocol initially described by Kramer et al. [8] and later utilized by Pierce et al. [9], Bracken et al. [10], and Lindo et al. [12]. Briefly, animals are sedated with a cocktail of anesthetics and tranquilizers (100 mg ketamine/10 mg xylazine/1 mg acepromazine/mL at 0.2 ml/kg) to reduce motion artifacts during MRI sessions. Intravenous contrast medium (0.25 mmol/kg of gadolinium chelate as ProHance; Bracco Diagnostics, Inc. Princeton, NJ, USA) was given to improve signal to noise, and each animal was placed inside the MRI unit (3 T Siemens Trios, Erlangen, Germany) with their back and legs supported in a natural position inside an eight-channel wrist coil (model HRW; Philips Medical Systems, Waukesha, WI, USA). Two localizing scans were obtained to locate anatomy and set the region of interest to produce serial images from L7 through C4. A sequence of echo gradient axial images was acquired during a 4.4-min interval. Images were saved as a DICOM series and transferred to a work station with commercial software (3D-Doctor, Able Software Corp, Lexington, MA, USA), which was used to review, manipulate, and measure the EAS.

### Histology

Following euthanasia, tissues were obtained. Cross-sectional blocks of rectal tissue beginning at the anal opening were embedded in gum on cork disks and snap frozen in liquid isopentane cooled by immersion in liquid nitrogen and stored at −70 °C. EAS was serially cut into 20-µm sections and was stained for succinate dehydrogenase in striated muscle fibers [15]. Areas of each stained section were traced with SigmaScan Pro software (Jandel Scientific Software, San Rafael, CA, USA), and volumes calculated by adding the areas of each section times the section thickness.

### Statistical Methods

Comparison of the two independent observers was made with Cronbach alpha for inter-rater correlation. Groups were compared using Student’s *t* test for parametric variables. Linear regression analysis examined the relationship of the EAS volume measured by MRI and the histological method, as well as the relationships between EAS volume and age, weight, and parity. Serial observations of EAS volumes were measured using ANOVA. A *p* value less than 0.05 was considered significant. The potential impact of the combination of pregnancy and aging to alter EAS volume was assessed using serial measurements in a cohort of four females that were imaged at less than 1 month of gestation, 1 to 4 days (mean of 3 days) postpartum, and 2 to 4 months postpartum. The 1- to 4-days postpartum imaging and the 2- to 4-months postpartum imaging were repeated after each of five pregnancies and vaginal deliveries, giving a total of 11 MRI sessions and image sets for each of four females. These data were examined using analysis of variance with a repeated measures design and Dunnett’s post hoc test from comparisons of EAS volume after each delivery with the initial pre-pregnancy value.

## Results

### External Anal Sphincter Volume Validation: Histology Versus Magnetic Resonance Imaging

**Fig. 1:**
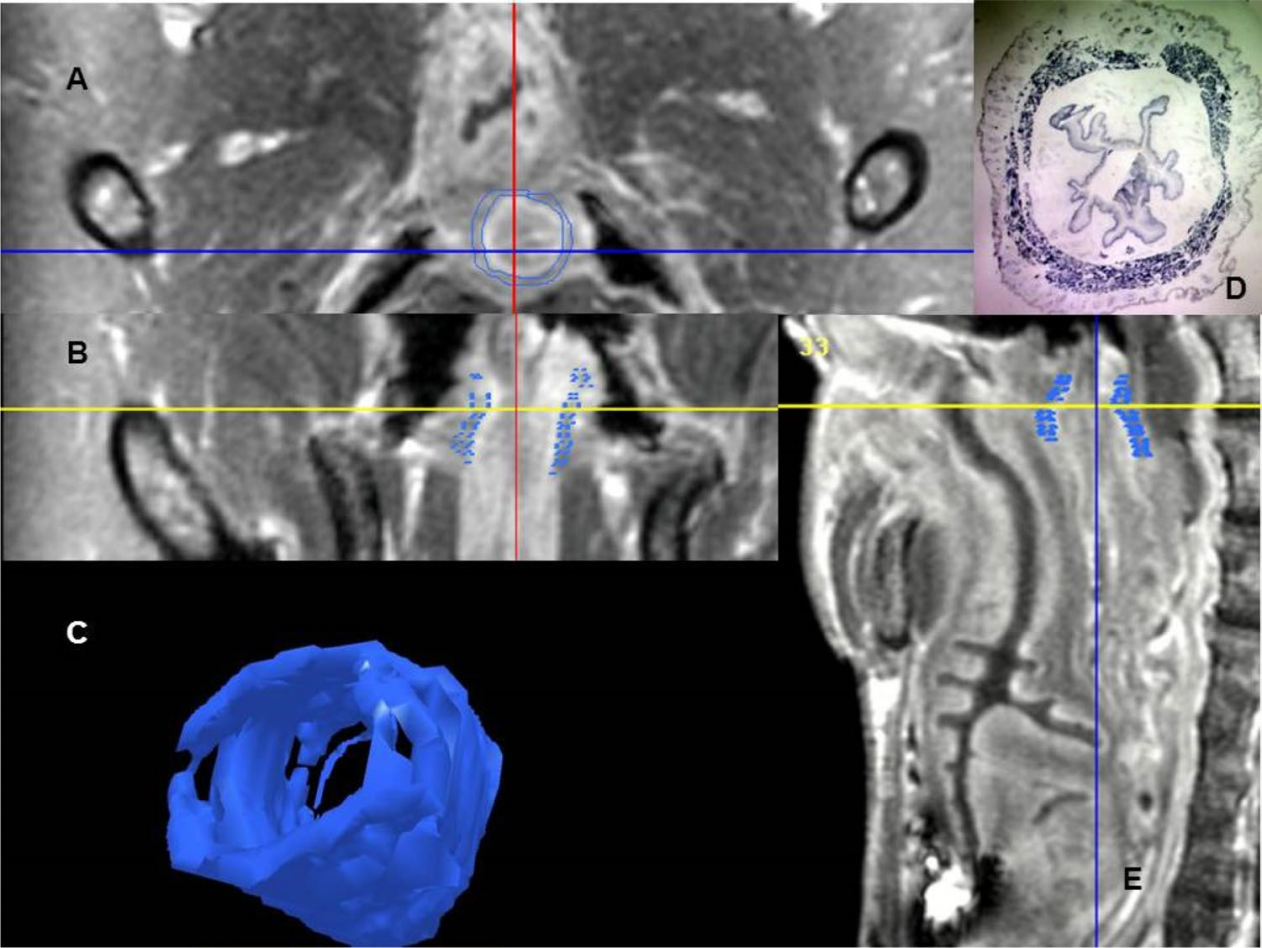
Magnetic resonance images (MRI), histology section, and three-dimensional model of the external anal sphincter from an 11-year-old nulliparous female (animal 10 in Table 1). The external anal sphincter is outlined in blue. **A** is an axial MRI. **B** is a coronal MRI. **C** is the three-dimensional model developed by tracing serial axial images. **D** is a stained 20-micron section corresponding to the axial image (**A**). **E** is the sagittal MRI

**Fig. 2:**
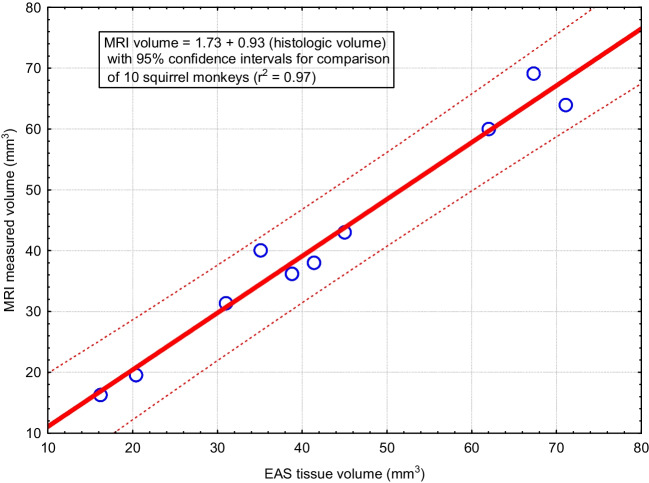
The mean of two independent observers measuring the external anal sphincter (EAS) with magnetic resonance imaging compares well with volumes derived from the sum of serial sections stained for striated muscle fibers of the EAS in serial sections from ten female squirrel monkeys

Two independent researchers used MRI to measure EAS volumes of ten squirrel monkeys. Figure 1 presents example MRI, a stained histology section, and a three-dimensional model prepared for one of these females. The volumes of the three-dimensional models were consistently measured by the two investigators (Cronbach alpha of 0.97 with 95% lower confidence limit of 0.92). Therefore, averages were used for the remaining analyses. MRI-measured volumes were compared with those from serial sections of the EAS in ten females (Fig. 2). Cronbach alpha for this comparison was 0.99, with a 95% lower confidence limit of 0.98 and a linear regression with R^2^ of 0.97 and *p* < 0.0001). The data set from the ten females is shown in Table 1. With this small sample of squirrel monkeys, no significant relationships between EAS volume measured either using tissue histology or MRI was observed for age, body weight, or parity (Table 2). Four of the ten females had POP based on visual inspection and descent of bladder observed with dynamic MRI [1, 9]. As symptoms of anal incontinence cannot be observed in this species, no relationship between EAS volume and continence could be evaluated. No externally visible anal opening lesions were seen. No difference in the average EAS volume either by histology (*p* = 0.07) or MRI (*p* = 0.35) was found to be related to the presence or absence of vaginally observed POP is this sample. The EAS is a small structure averaging 42 mm^3^ (range 20 to 70 mm^3^) in the squirrel monkey female, based on the MRI measurement.

**Table 1:** Characteristics and measurements of external anal sphincter (EAS) in 10 females using both magnetic resonance imaging and histological methods

Animal	Characteristic					
	Age (years)	Weight (g)	Parity	Pelvic organ prolapse	MRI EAS volume (mm^3^)	Histological EAS volume (mm^3^)
1	17.8	707	3	No	36.2	38.8
2	12.8	793	4	Yes	19.6	20.4
3	13.8	721	3	No	64.0	71.1
4	12.8	703	8	Yes	43	45
5	11.8	699	5	Yes	38	41.4
6	11.4	623	5	No	60	62
7	9.8	774	1	No	69.1	67.3
8	6.7	658	1	Yes	31.4	31
9	6.9	770	3	No	16.3	16.2
10	11.5	949	0	No	40.1	35.1
Mean ± SD	11.5 ± 3.3	740 ± 90	3 ± 2	4/10 (40%)	41.8 ± 17.9	42.8 ± 18.9

**Table 2:** Correlations of age, weight, parity to external anal sphincter (EAS) volume for 10 females

Characteristic	MRI EAS volume		Histology EAS volume	
	Correlation coefficient	*p* value	Correlation	*p* value
Age	0.20	0.57	0.27	0.45
Weight	−0.19	0.60	−0.29	0.41
Parity	−0.04	0.91	0.05	0.88

*MRI* magnetic resonance imaging

### Serial Measurements of External Anal Sphincter Using Magnetic Resonance Imaging

**Fig. 3:**
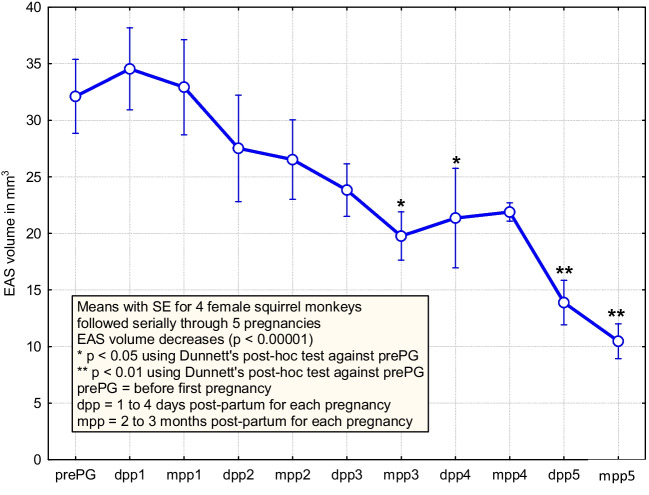
Serial measurements of external anal sphincter (EAS) volume using magnetic resonance imaging (MRI) in four female squirrel monkeys undergoing five pregnancies with spontaneous vaginal deliveries. Animals were examined by MRI prior to the first pregnancy, within 5 days post-partum of each delivery and again 2 to 4 months post-partum after each delivery. The EAS volume decreased gradually to half its initial size after five deliveries

Data from concurrent study of serial MRI measurements show a gradual decrease in EAS volume over time as age and parity increase from the cohort of 4 breeding females (*p* < 0.00001; Fig. 3). This plot uses the average of two independent observers and demonstrates the value of using serial observations for a small sample of animals. In a series using 4 animals, time, which includes both the impact of aging and parity, was found to be associated with a decrease in EAS volume, whereas the results from 10 euthanized animals with single observations as presented in Tables 1 and 2 did not demonstrate these effects.

## Discussion

This study showed that EAS is a small structure averaging 42 mm^3^ (range 20 to 70 mm^3^) in the squirrel monkey female. EAS volume can be reliably traced and measured using MRI, as demonstrated by similarity between two independent observers, and between MRI measurements and measurements obtained from direct histological tissue. In a series of 10 euthanized animals, no associations of EAS volume with age, parity, body weight or presence of pelvic support defects were detected. However, using MRI methods with increased power of within animal serial observations, time (which included the impact of age and parity) was found to be associated with a decrease in EAS volume as might be anticipated based on human patient observations of EAS injury related to childbirth and aging. This demonstrates the benefit of using a validated MRI method for serial measurements. Vaginal delivery may cause injury to the pelvic nerve and cause muscle injury and atrophy as observed by MRI in this cohort of breeding squirrel monkeys. Alternatively, aging may lead to sarcopenia and/or a combination of both factors in these breeding females may contribute to the observed changes. Of course, no instrumentation of delivery was performed, so this is not a factor using the squirrel monkeys as a model species. Squirrel monkeys have a relatively soft stool owing to their diet and fluid consumption and short, large intestine. This is likely an adaptation to living in a relatively wet environment. This finding suggests that the decrease in EAS volume is more likely due to serial pregnancies and/or aging and less likely due to other factors such as constipation. A prospective randomized trial would be needed to separate the impact of pregnancy and vaginal delivery from that of aging alone or planned cesarean delivery prior to labor.

In our institution, we have been studying pelvic floor disorder using squirrel monkeys for many years. Squirrel monkey females deliver large infants; newborns are about 14% of their mothers’ body weight. This is comparable with a 130-pound woman delivering an 18-pound baby. Perinatal and maternal mortality are high in squirrel monkeys [3]. They have a 152-day gestation, and infants are born with a relatively mature brain enclosed in a skull with closed suture joints. Hence, females are at a high risk for developing pelvic organ prolapse. EAS injury in humans is associated with pregnancy, delivery, and aging [15, 17, 18]. The frequency of EAS injuries and the potential mechanisms for their development in squirrel monkeys have not been evaluated.

The EAS is a flat plane of muscular fibers measuring about 10 cm in the human. The contraction of EAS keeps the anal canal closed. Vaginal delivery can be traumatic to the EAS, resulting in its disruption and leading to fecal incontinence in later life. Traditionally, EAS disruption is visualized using endo-anal ultrasound and evaluated during clinical examination. Quantitative three-dimensional ultrasound has been used to measure the anal sphincter volume [16]. Cornella et al. used three-dimensional MRI to reconstruct EAS and measure EAS volume [20]. They found EAS to be funnel-shaped, being narrower at the caudad end, widening in the cephalad direction, and elongating in the anterior–posterior diameter [20]. They also reported an association of EAS volume with pressure generation. Thus, as symptoms of anal incontinence cannot be assessed in the squirrel monkey as a model species, EAS volume might be a use indicator or marker.

Magnetic resonance imaging has been used to measure pelvic floor muscle volumes in female squirrel monkeys to describe changes related to pregnancy, method of parturition, and aging [8, 10, 12]. A histological method for measurement of EAS muscle mass has been described in a rat model of childbirth injury to EAS [15]. Denervation and re-innervation of the EAS after childbirth can lead to alteration in muscle function that contributes to incontinence [16] and MRI measurements of volume are associated with sphincter function in patients [20]. Boyle et al. showed that anal resting tone decreased with age and childbirth in humans [14]. They also observed that instrumented delivery is detrimental to the structure and function of EAS [14]. The impact of planned cesarean section delivery of EAS is difficult to assess in humans without randomization. However, randomized studies can be performed in the squirrel monkey as a model species [12].

In conclusion, we were able to demonstrate using a reliable MRI method, that serial measurements over time, including the impact of pregnancies and aging, are associated with decreased EAS volume in this species.

## Data Availability

Data files can be obtained though contact to one of the authors: Thomas J. Kuehl at tjkdata@peoplepc.com.
